# Bone Mineral Changes in Spine and Proximal Femur in Individual Obese Women after Laparoscopic Sleeve Gastrectomy: A Short-Term Study

**DOI:** 10.1007/s11695-012-0654-8

**Published:** 2012-05-04

**Authors:** Wojciech Pluskiewicz, Marek Bužga, Pavol Holéczy, Ladislav Bortlík, Vít Šmajstrla, Piotr Adamczyk

**Affiliations:** 1 Metabolic Bone Diseases Unit, Department and Clinic of Internal Diseases, Diabetology and Nephrology, Medical University of Silesia in Katowice, ul. 3-Maja 13/15, 41-800 Zabrze, Poland; 2 Department of Physiology, Medical Faculty, University of Ostrava, Ostrava, Czech Republic; 3 Department of Surgical Disciplines, Medical Faculty, University of Ostrava, Ostrava, Czech Republic; 4 Bone Densitometry Laboratory, Bormed, Ostrava, Czech Republic; 5 Department and Clinic of Pediatrics, Medical University of Silesia, Katowice, Poland

**Keywords:** Bone mineral density, Obesity, Laparoscopic sleeve gastrectomy, Women

## Abstract

**Background:**

The aim of the study was to establish longitudinal bone changes in obese women after laparoscopic sleeve gastrectomy (LSG).

**Methods:**

Twenty-nine women at baseline mean age of 40.41 ± 9.26 years and with mean body mass index (BMI) of 43.07 ± 4.99 kg/m^2^ were included in a 6-month study. Skeletal status at hip [femoral neck (FN) and total hip (TH)] and spine was assessed at baseline, as well as in 3 and 6 months after surgery. Body size was measured at baseline and follow-up (weight, height, BMI, and waist).

**Results:**

Baseline body weight was 117.5 ± 18.4 kg. The mean body weight and BMI decreased by 17.9 % during the first 3 months after surgery to obtain 28.4 % after 6 months. At 6 months, BMD decreased significantly for spine by 1.24 %, FN 6.99 %, and TH 5.18 %. The changes after 3 months in individual subjects showed that, in the majority of subjects, FN and TH BMD decreased significantly (in 52 % and 69 % of subjects, respectively), and in 24 % loss of BMD was found at the spine. After 6 months, the corresponding, significant decreases in individual subjects were found in 72 %, 86 %, and 38 % of woman, respectively. Those with a significant loss of FN BMD tended to lose more weight (30 ± 9.47 versus 23.25 ± 6.08 kg, *p* = 0.061) than others; women with a significant decrease of FN BMD lost more weight than those with no such decrease (30.43 ± 8.07 versus 15 ± 1.91 kg).

**Conclusion:**

LSG proved efficient for body weight reduction, however, with a parallel decline in bone mineral density.

## Introduction

Obesity is a growing medical and social problem worldwide. Several health complications are common among subjects with obesity, especially when the body mass index (BMI) exceeds 40 kg/m^2^. In a significant part of obese subjects, modifications of lifestyle and pharmacotherapy fail to bring about expected, significant weight loss. Bariatric surgery has proven to be the most effective way of treatment for morbidly obese women and men [1] and may be a cost-effective alternative treatment in morbid obesity [2]. The prevalence of weight-related morbid conditions decreases significantly after bariatric surgery [3]. Many different surgical techniques are used in obese patients, including pure restriction surgery (gastric banding, vertical banded gastroplasty, and sleeve gastrectomy) and malabsorption surgery, either with or without associated restrictions [Roux-en-Y gastric bypass –(RYGBP), duodenal switch, and biliopancreatic diversion]. However, bariatric surgery, while being effective for weight loss, may also result in several health complications, including bone metabolism and status [1]. In several already published reports, the role of this treatment method was approached from the bone health perspective [4–13]. In some studies, BMD changes were followed up in subjects after RYGBP [4–9], biliopancreatic diversion [10, 11], or gastric banding [12, 13]. RYGBP, a very efficient technique, promotes significant changes in gastrointestinal anatomy, leading to nutritional and metabolic balance [14, 15].

Lately, an increasing interest has been observed regarding other surgical techniques used in bariatric patients. For example, laparoscopic sleeve gastrectomy (LSG) is a technique which changes the gastrointestinal status. In several recently published studies, LSG was compared with RYGBP, and both procedures led to comparable weight loss, associated with the resolution of the metabolic syndrome [16–19]. One may only expect that the bariatric procedure is associated with fewer disturbances regarding bone metabolism and status. To our knowledge, the effects of this surgical technique on bone metabolism and status were studied in only one small study [20]. The aim of the current study was then to establish longitudinal bone changes in individual women after LSG.

## Materials and Methods

Twenty-nine (29) obese women at mean baseline age of 41.1 ± 9.78 years were included into the study. They were recruited from the Department of Obesitology at the Vitkovice Hospital in Ostrava, the Czech Republic, and submitted to bariatric surgery in order to have their body weight reduced. Table 1 presents clinical characteristics of the studied women. The majority of them were at premenopausal, and four (13.3 %) were at postmenopausal age. None of the examined women presented with concomitant diseases, which could have potentially influenced their bone metabolism. The subjects were operated during the period of September/December 2010, as part of an open prospective clinical pilot study monitoring the metabolic response of lipid and bone tissue, following LSG. After the surgery, patients enrolled in the study were not included in subsequent rehabilitation exercises. The rate of physical activity has been studied neither in terms of aerobic exercise nor strength training. During the observation period of 6 months, four women were excluded from the study (reoperation and noncompliance).

**Table 1:** Clinical characteristics, body size, and densitometric variables at baseline and after 3 and 6 months, expressed as means and SDs

Variable	Baseline	After 3 months		After 6 months		
	Mean ± SD	Mean ± SD	*p* value vs. baseline examination	Mean ± SD	*p* value vs. baseline examination	*p* value vs. examination after 3 months
Weight [kg]	117.5 ± 18.4	96.44 ± 17.76	<0.000001	89.2 ± 16.06	<0.000001	<0.000001
Excess body weight [%]	92.15 ± 23.4	57.6 ± 24.4	<0.000001	45.7 ± 21.8	<0.000001	<0.000001
Height [cm]	164.9 ± 7.39	164.98 ± 7.4	NS	164.98 ± 7.4	NS	NS
BMI [kg/m^2^]	43.01 ± 4.99	35.31 ± 5.28	<0.000001	32.56 ± 4.75	<0.000001	<0.000001
Waist [cm]	112.7 ± 10.51	98.44 ± 10.37	<0.000001	93.96 ± 9.14	<0.000001	<0.0001
Hips [cm]	136.3 ± 11.64	121.9 ± 12.91	<0.000001	116.9 ± 12.21	<0.000001	<0.00001
Spine BMD [g/cm^2^]	1.163 ± 0.15	1.165 ± 0.15	NS	1.148 ± 0.16	<0.05	<0.001
T-score	1.05 ± 1.39	1.07 ± 1.41	NS	0.92 ± 1.42	<0.05	<0.001
Z-score	1.45 ± 1.51	1.49 ± 1.53	NS	1.33 ± 1.52	<0.05	<0.001
FN BMD [g/cm^2^]	0.988 ± 0.14	0.94 ± 0.12	<0.00001	0.917 ± 0.11	<0.000001	<0.0001
T-score	1.26 ± 1.29	0.84 ± 1.1	<0.00001	0.59 ± 1.01	<0.000001	<0.0001
Z-score	1.68 ± 1.25	1.27 ± 1.11	<0.00001	1.04 ± 1.02	<0.000001	<0.0001
TH BMD [g/cm^2^]	1.135 ± 0.12	1.095 ± 0.12	<0.000001	1.07 ± 0.11	<0.000001	<0.0001
T-score	1.57 ± 0.98	1.25 ± 0.99	<0.000001	1.11 ± 0.95	<0.000001	<0.001
Z-score	1.85 ± 1.01	1.55 ± 1.05	<0.000001	1.39 ± 1.03	<0.000001	<0.001

### Surgery

All the women underwent LSG as a restrictive bariatric procedure, which involves subtotal gastric resection of the fundus and body to create a long, tubular gastric conduit, constructed along the lesser curve of the stomach. It was originally described as the first stage bariatric procedure, followed by Roux-Y gastric bypass or biliopancreatic diversion with duodenal switch in high-risk patients. However, given the benefits of low risk, simple surgery with possible surgical revision when required, more and more bariatric surgeons accept it as the primary bariatric procedure.

The selection for surgical treatment was made in accordance with the IFSO guidelines, i.e., in individuals with BMI >40 kg/m^2^ or of BMI >35 /m^2^ with comorbidities. The study was approved by the Ethical Committee at the Medical Faculty of the Ostrava University. The patients were operated at the Bariatric Center of the Vitkovice Hospital in Ostrava, the Czech Republic. The studied women were subject of long-term observations, and all the procedures, as described in the study report, were performed at baseline (before the surgery) and after 3 and 6 months.

The operation was performed in general anesthesia at supine position. Pneumoperitoneum was made using a Veress needle. A 30 ° laparoscope was used. The area of diaphragmatic hiatus was revised in detail. Should hiatus hernia have been found, hiatoplasty was performed. The subsequent step involved mobilization of the greater curvature of the stomach, which started proximally, 4 cm from the pylorus. That step was made by an ultrasound dissector (Harmonic Ace, Johnson and Johnson, Cincinnati, OH, USA). The mobilization stretched as far as to the left diaphragmatic crus. Following satisfactory mobilization, the stomach was longitudinally resected. The resection was done by means of an Echelon endoscopic stapler (Johnson and Johnson, Cincinnati, OH, USA) with blue cartridges of 60 mm length. The size of the left stomach portion was subject to anatomic border—the end of the short gastric vessel from the lesser curvature of the stomach. In that phase of the operation, neither calibration bougie was applied nor reinforcement of the resection line was used.

### Body Size

In all the women, the following body size measurements were obtained: body weight, body height, abdominal waist circumference (halfway last rib and the iliac crest), and hip circumference. In order to determine the weight and height, an anthropometric calibrated meter was used. Body weight was measured in volunteers in their underwear with precision to 0.2 kg. The accuracy for height measurements was 0.1 cm. All the measurements were performed by a nurse trained in anthropometric measurements.

### Bone Densitometry

Bone densitometry was performed using a Hologic Discovery W machine (USA). Spine and proximal femur [femoral neck (FN) and total hip (TH)] bone mineral density (BMD) was measured. The machine was calibrated, according to the manufacturer's recommendations. A precision error was also established for the densitometer. CV% was established for that device, and for FN, TH, and spine, a series of measurements was performed in 15 patients with repositioning. CV% values were calculated, according to the following formula: CV% = SD/mean × 100 %. CV% values were 0.66 %, 1.49 %, and 0.74 % for spine, FN, and TH, respectively.

### Statistics

All the analyses were performed using the Statistica program (StatSoft, Inc., Tulsa, OK, USA). Descriptive statistics was presented as mean values, standard deviations, and value ranges. The distribution of analyzed data was checked by the Shapiro–Wilk test. The differences between results, obtained at baseline and at follow-up, were established, using Student's *t* test for dependent samples. A correlation analysis at baseline and follow-up was done by Pearson's or Spearman's test, whichever was appropriate. Individual differences between baseline and follow-up measurements, expressed as Δ of the measured variables, were established, and a correlation analysis was performed for them, using also Pearson's or Spearman's correlation test, whichever was appropriate. In order to follow reliable changes of densitometric variables in individual patients, the least significant change (LSC) was calculated. The LSC, or a critical difference, denotes the minimal difference between two successive results in an individual that can be considered to reflect a real change. The LSC was calculated, using the following formula: RMS_CV × 2 × 1.41, which represent a statistical difference at the 95 % confidence level [21]. *P* value lower than 0.05 was considered as statistically significant.

## Results

### Changes in Body Size

**Fig. 1:**
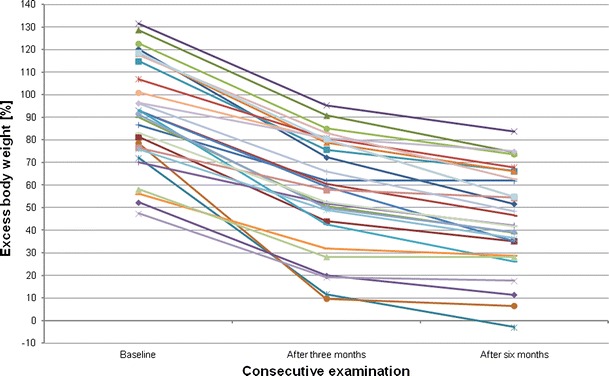
Individual values of excess body weight (expressed as percentage of ideal weight) during the period of observation

All the measured body size variables were significantly lower after 3 and 6 months (Table 1). Table 2 shows differences, expressed as Δ in body size and densitometric variables, recorded over the observation period. In general, a decrease was observed mostly during the first 3 months. For example, total body weight loss during the first 3 months was by 17.9 %, falling to 6.2 % during another 3 months. Figure 1 presents a decrease in excessive body weight after 3 and 6 months.

**Table 2:** Changes in body size and densitometric variables after 3 and 6 months, expressed as means and SDs

Variable	Δ (difference between baseline and 3 months)	Δ (difference between baseline and 6 months)
Weight [kg]	−21.1 ± 7.21	−28.37 ± 9.15
BMI [kg/m^2^]	−7.76 ± 2.7	−10.42 ± 3.34
Waist [cm]	−14.27 ± 6.73	−18.75 ± 8.27
Hips [cm]	−14.3 ± 6.95	−19.37 ± 7.44
Spine BMD [g/cm^2^]	0.0023 ± 0.02	−0.014 ± 0.03
FN BMD [g/cm^2^]	−0.046 ± 0.04	−0.072 ± 0.046
TH BMD [g/cm^2^]	−0.039 ± 0.02	−0.059 ± 0.03

### Bone Mineral Changes—Mean Values

The mean BMD values decreased significantly for spine, FN, and TH over the observation period. Densitometric data are presented in Table 1. During the first 3 months of observation, the mean FN and TH BMD values decreased significantly by 4.45 % and 3.5 %, respectively, while the spine BMD remained stable. Further, some decrease was observed for all the observed skeletal sites. In general, a decline for the proximal femur was more distinct during the first 3 months. The mean spine BMD value decreased significantly by 1.24 %, FN BMD by 6.99 %, and TH BMD by 5.18 % over the observation period. Taking into account the whole period of observation, the spine BMD decrease was significantly smaller than BMD decrease for FN (*p* < 0.00001) or TH (*p* < 0.00001), and the decrease in FN BMD was significantly bigger than for TH (*p* < 0.01). The values of T-score and Z-score for spine, FN, and TH were significantly lower at follow-up (*p* < 0.000001; see Table 1).

### Bone Mineral Changes—Results in Individual Patients

An analysis was performed with regards to BMD changes in individual subjects. The analysis of the mean changes in the whole group allowed assessing general trends, but when a longitudinal observation is carried out, one should also take into consideration the precision of used devices. That analysis enabled us to find out where (i.e., in which women) BMD values decreased more than LSC values during 3 and 6 months.

**Fig. 2:**
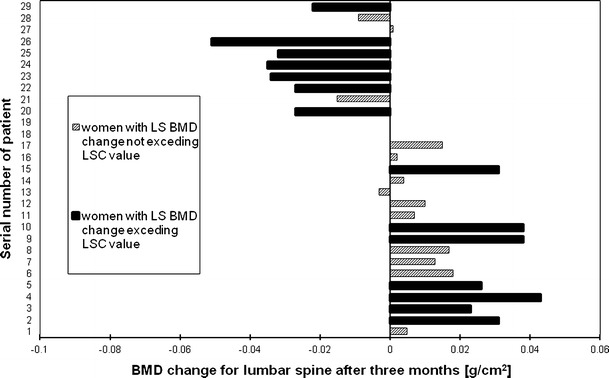
Longitudinal BMD change for lumbar spine during the first 3 months of observation in individual patients. *LS BMD* lumbar spine bone mineral density, *LSC* least significant change

**Fig. 3:**
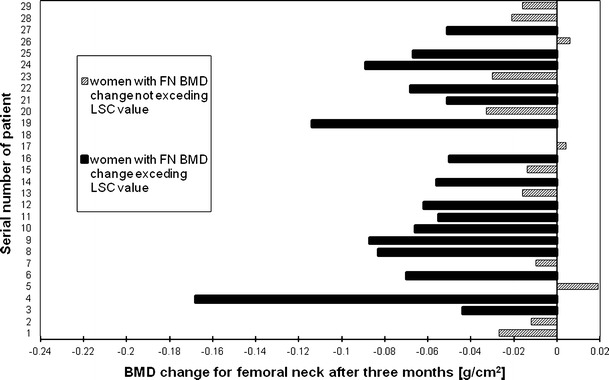
Longitudinal BMD change for femoral neck during the first 3 months of observation in individual patients. *FN BMD* femoral neck bone mineral density, *LSC* least significant change

**Fig. 4:**
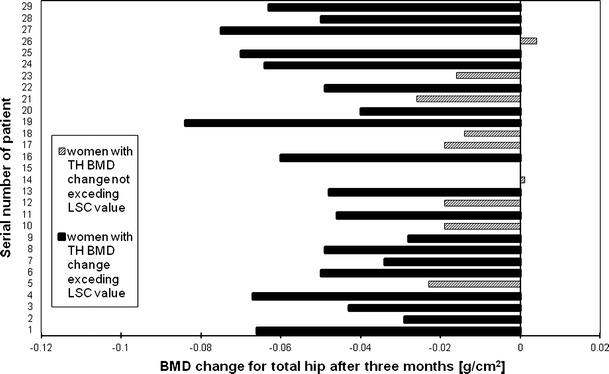
Longitudinal BMD change for total hip during the first 3 months of observation in individual patients. *TH BMD* total hip bone mineral density, *LSC* least significant change

**Fig. 5:**
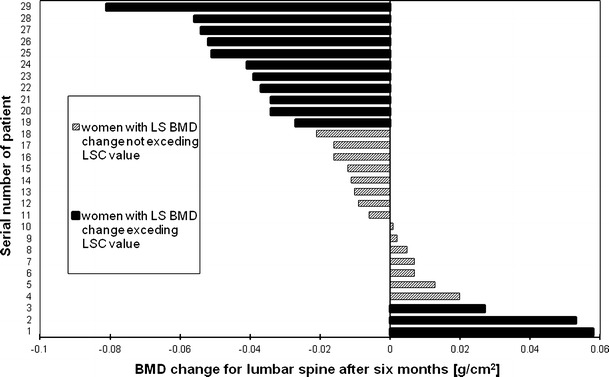
Longitudinal BMD change for lumbar spine during 6 months of observation in individual patients. *LS BMD* lumbar spine bone mineral density, *LSC* least significant change

**Fig. 6:**
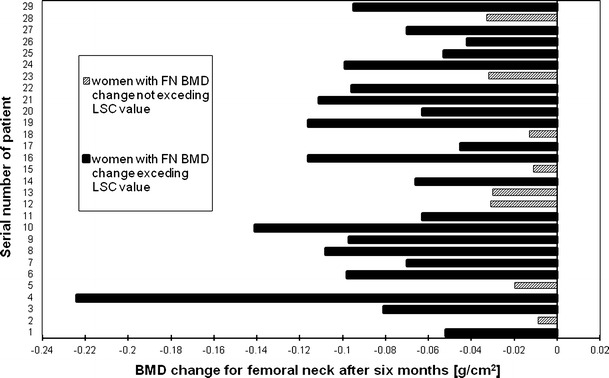
Longitudinal BMD change for femoral neck during 6 months of observation in individual patients. *FN BMD* femoral neck bone mineral density, *LSC* least significant change

**Fig. 7:**
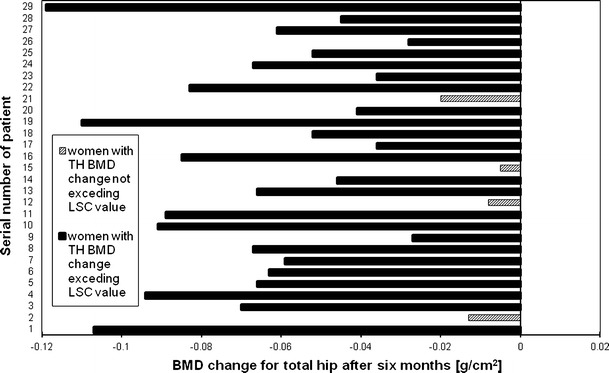
Longitudinal BMD change for total hip during 6 months of observation in individual patients. *TH BMD* total hip bone mineral density, *LSC* least significant change

**Fig. 8:**
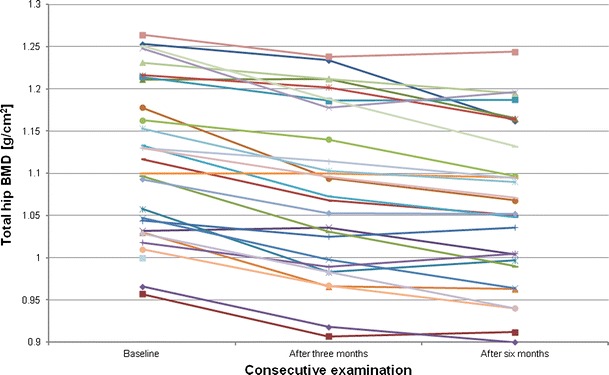
Individual values of BMD for total hip during the period of observation. *BMD* bone mineral density

Figures 2, 3, 4 and 5, 6, 7 presented BMD changes in individual patients after 3 and 6 months, respectively. In all the figures, the results of individual patients are presented in the same order. The changes, observed after 3 months in individual subjects, confirmed that, in the majority of subjects, FN and TH BMD decreased significantly (52 % and 69 %, respectively), and only in 24 %, regarding BMD at the spine. Spine BMD increase was noted in 24 % of the studied women. Figure 5 presents data for spine BMD after 6 months; in 11 patients, BMD decreased (38 %); in 15, it was stable (52 %); and in 3, it increased (10 %). In the majority of the studied women, FN BMD decreased (21 patients, 72 %; Fig. 6), and regarding TH BMD, a decrease was observed in 25 women (86 %; Fig. 7). Figure 8 shows differences of TH BMD, expressed in grams per square centimeter.

We also assumed that in women with a significant decrease in BMD, there should be a more pronounced decrease in body weight as well. Those who lost FN BMD tended to lose more weight (30.0 ± 9.47 versus 23.25 ± 6.08 kg, *p* = 0.061) than those who did not, and in women with a significant decrease of TH BMD, there was more weight lost than in those with no such a decrease (30.43 ± 8.07 versus 15 ± 1.91 kg). Regarding the changes in spine BMD, no significant differences were observed in body weight.

### Correlation Analysis Between Body Size and BMD

A correlation analysis between BMD baseline values and body size showed a significant relationship between FN BMD with weight (*r* = 0.39, *p* < 0.05) and spine BMD with height (*r* = 0.55, *p* < 0.01). The same analysis after 6 months revealed the following significant correlations: spine BMD with height (*r* = 0.54, *p* < 0.01) and FN BMD with height (*r* = 0.47, *p* < 0.05).

### Correlation Analysis Between Changes in Body Size and Changes in BMD—3 months

Δ values for body size after 3 months were correlated with Δ values of densitometric variables and showed the following results: Δ for TH BMD correlated with Δ for body size (BMI, *r* = 0.44, *p* < 0.05; waist 1, *r* = 0.45, *p* < 0.05; waist 2, *r* = 0.53, *p* < 0.01; hips, *r* = 0.59, *p* < 0.01). Δ for FN and spine BMD did not correlate with changes in body size.

### Correlation Analysis Between Changes in Body Size and Changes in BMD—6 months

Δ values for body size after 6 months were correlated with Δ values of densitometric variables and showed the following results: Δ for FN BMD correlated with Δ for weight (*r* = 0.51, *p* < 0.01), Δ for BMI (*r* = 0.51, *p* < 0.01), and Δ for hips (*r* = 0.46, *p* < 0.05); Δ for TH BMD correlated with all Δ for body size (weight, *r* = 0.48, *p* < 0.01; BMI, *r* = 0.54, *p* < 0.01; waist 1, *r* = 0.37, *p* < 0.05; waist 2, *r* = 0.47, *p* < 0.05; hips, *r* = 0.58, *p* < 0.01). Δ for spine BMD did not correlate with Δ for body size.

### Bone Mineral Changes—Normal Value, Osteopenia, and Osteoporosis

We also established a number of subjects with normal values, osteopenia, and osteoporosis for measured skeletal sites at baseline and follow-up. In all the women at baseline, FN and TH BMD values were within normal limits (T-score above −1.0), and in two women, T-score was in the range of osteopenia (T-score between −1.0 and −2.5). At follow-up regarding spine BMD, two subjects remained osteopenic, two presented with T-score for FN below a threshold of −1.0, and for all the women, T-score for TH was normal.

## Discussion

To our knowledge, the reported study is the first longitudinal observation of BMD changes in patients after laparoscopically performed sleeve gastrectomy. Despite the short duration of the study, BMD decrease was demonstrated in various skeletal sites, but we consider that the most important clinical findings concerned the changes observed in individual patients. In a longitudinal observation, the essential role is played by precision of the used method and, therefore, a concept of the LSC was included in our analysis. Therefore, we were able to reliably find out real bone losses. In our opinion, such methodology was not used before with regards to patients after bariatric surgery. In some studies, concerning bone changes in various groups of patients, such an interpretation of results was applied [22–24]. An early decrease in BMD occurred after 3 months from the surgery for the proximal femur in the majority of patients and only in one fourth for the spine. For the whole period of observation, the proximal femur BMD decreased significantly in the majority of the studied patients, and in less than half of them, spine bone loss was noted.

In obese subjects after bariatric surgery, the most important factor, influencing bone status, is probably the loss of body weight. Surgical intervention in bariatric patients should result in decreased body size and LSG, performed in our patients, confirmed the efficacy of the method. We assumed that in women with a significant decrease in BMD, a more pronounced decrease in body weight should be present. That thesis was supported at borderline significant level for FN changes and in total for TH bone changes. In Figs. 1 and 8, changes are shown in body weight and TH BMD, respectively, and it may be seen that the pattern of longitudinal changes is similar.

Only one small study compared the results of patients, operated by SG and RYGBP procedure [20]. Fifteen women with morbid obesity were included, eight after SG and seven after RYGBP, their mean age of 47.8 ± 9 years and the mean BMI of 43.3 ± 3.4 kg/m^2^. Densitometry of lumbar spine, femur, and distal radius was performed before and 12 months after surgery. A significant bone mass loss was observed in patients after SG and RYGBP surgery in the lumbar spine and hip, while no differences were observed in the radial status. The percentage of BMD loss was less in the spine and femur after SG than with RYGBP, although it did not reach statistical significance (4.6 % and 6.3 %, respectively). The authors concluded that SG caused less, although not significant, bone mass loss, compared to RYGBP. In the current study, we noted a clearly smaller decrease for spine but greater for FN.

We demonstrated that in obese women with the mean baseline BMI of 43 kg/m^2^, successful results of body size reduction were possible. The mean decrease of 28 % in baseline body weight was noted in our group, which is comparable to the results presented by other authors, after 1 year from surgery with gastric banding; four of them noted a similar weight loss, ranging from 23.3 % [25] to 25 % [12, 13, 26], and only Vilarrasa et al. observed a greater weight loss of 33.7 % [8]. Some authors presented weight loss after 6 months, and their results of 13 % [4] and 16 % [13, 25] were smaller than noted in the reported study.

Parallel to expected changes in body weight, also noted was a significant decrease for spine and proximal femur BMD. The most significant decline in BMD was observed for the FN, followed by TH and spine. Relationships were also established between changes in body size and BMD (both expressed as Δ), and a bigger decrease in body size was connected with a more pronounced decline in BMD values except for those in the spine. Interestingly enough, only some correlations of body size with densitometric variables were noted at baseline. The decline in FN was most pronounced, which is comparable to the data given by some of the other authors [8, 26]. Some authors observed a stable [12] or even increased value of spine BMD [13, 25]. In the reported longitudinal study, the majority of studied women presented BMD remaining within normal values, but further observation is necessary in order to verify the thesis that a decline of BMD may lead to the development of osteoporosis. In the current study, regarding the baseline and follow-up spine BMD values, only in two subjects was osteopenic, and in two patients, T-score for FN was below a threshold of −1.0. In another study [8], the number of subjects with developing low bone mass a year after surgery was bigger: for FN from 1.6 % at baseline to 16.1 % at follow-up, and for spine, from 9.6 % to 19.3 %. Recently, fewer nutrient deficiencies are observed after LSG than after laparoscopic Roux-Y gastric bypass [19, 27], which supports the clinical value of SG as a method of surgical treatment of bariatric patients. Also, other recent studies present data showing that SG is a valuable method in obese patients, due to several reasons, including cholesterol profile [17], diabetes [18], and weight loss [19].

In a prospective observation, one should take into consideration the precision of used methods; especially, when the differences between baseline and follow-up values are not big, a precise evaluation of results in individual patients may provide important clinical data. Theoretically, a bigger weight loss should cause a more significant decrease in BMD. In our study, we proved that a greater decline in body weight correlated borderline significantly with a smaller decrease in FN BMD and, significantly, with TH BMD decrease. That observation suggests that weight loss plays an essential role in bone loss after bariatric surgery.

Our study has got several limitations: only women were observed, the follow-up was short, and the results of different surgical techniques were not directly compared. The study design did not include other factors potentially influencing bone metabolism and status (bone markers, vitamin D, and adipokines). However, irrespective to the pathophysiology of changes in bone metabolism, a significant decrease in bone densitometric variables was observed in a considerable part of studied women, especially for the proximal femur.

In conclusion, laparoscopy sleeve gastrectomy proved as an efficient method to decrease body weight with a parallel decline in bone mineral density.
